# Bromelain Immobilized onto Clay-Carboxymethylcellulose Composites for Improving Nutritive Value of Soybean Meal

**DOI:** 10.1021/acsabm.4c00392

**Published:** 2024-07-18

**Authors:** Kanlayanit Pimcharoen, Pakorn Opaprakasit, Yodying Yingchutrakul, Nattapon Simanon, Chutikarn Butkinaree, Darawan Yuttayong, Ramawadee Hompa, Lapporn Vayachuta, Panida Prompinit

**Affiliations:** † School of Integrated Science and Innovation, Sirindhorn International Institute of Technology (SIIT), Thammasat University, Pathum Thani 12121, Thailand; ‡ National Center for Genetic Engineering and Biotechnology, National Science and Technology Development Agency (NSTDA), Khlong Luang, Pathum Thani 12120, Thailand; § Aquatic Animal Feed Research and Development Division, Department of Fisheries, Ministry of Agriculture and Cooperatives, Bangkok 10900, Thailand; ∥ National Nanotechnology Center (NANOTEC), National Science and Technology Development Agency (NSTDA), Khlong Luang, Pathum Thani 12120, Thailand

**Keywords:** Carboxymethyl cellulose, bromelain, immobilization, ionotropic gelation, soybean meal

## Abstract

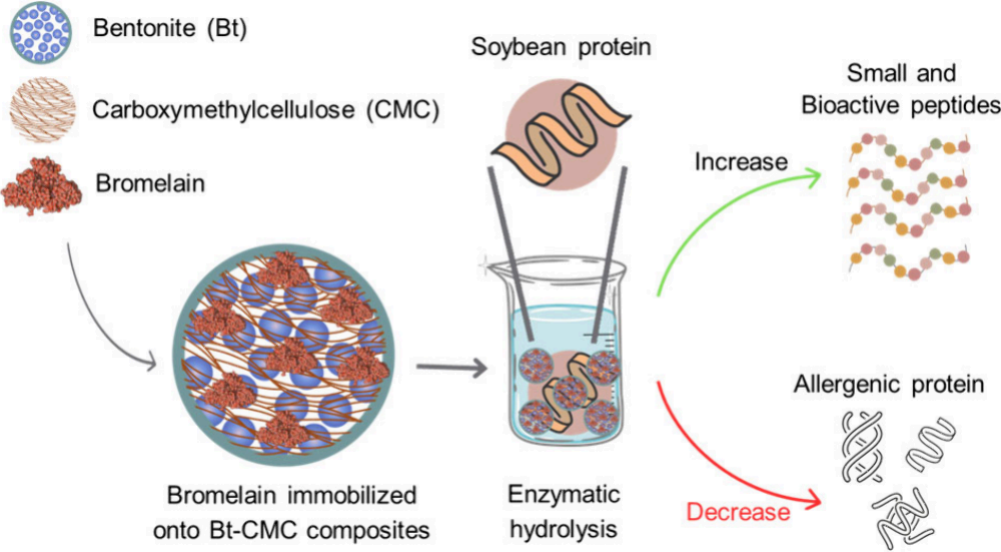

Improvement of nutritional value and reduction of antinutritional factors (ANFs) of soybean meal (SBM) for animal feed applications could be achieved by using bromelain immobilized onto bentonite (Bt)-carboxymethylcellulose (CMC) composites. The composite with mass ratio between CMC to calcium ion (Ca^2+^) at 1:20 provided the highest enzyme activity, immobilization yield higher than 95%, with superior thermal and storage stabilities. Performance of the immobilized bromelain for soybean protein hydrolysis was further studied. The results showed that at 60 °C, the immobilized bromelain exhibited the highest efficiency in enzymatic hydrolysis to release free alpha amino nitrogen (FAN) as a product with high selectivity and to effectively reduce SBM allergenic proteins within 30 min. In conclusion, immobilization of bromelain onto Bt-CMC composites leads to stability enhancement of the enzyme, enabling effective improvement in SBM quality in a short treatment time and showing great potential for application in animal feed industries.

## 1 Introduction

Soybean meal (SBM) is the most common plant-based protein source for animal feed because of its availability, low cost, favorable palatability, and balanced amino acid profile.^1,2^ However, it contains several antinutritional factors (ANFs) such as trypsin inhibitors, lectins, phytate, and saponin. Moreover, glycinin and β-conglycinin, two major allergenic proteins in SBM, can induce an allergic immune response, leading to abnormal morphology of the small intestine and diarrhea in animals.^3^ These parameters reduce the utilization and digestibility of soybean protein, which leads to impaired growth performance. Therefore, several methods have been attempted to eliminate the ANFs and allergenic proteins as well as upgrade its feeding value. These methods include ethanol extractions,^4,5^ microbial fermentation,^6,7^ and enzymatic hydrolysis.^8−11^ Among these approaches, treatment of SBM with enzyme offers more advantages over the others in terms of remarkable regioselectivity and stereoselectivity in reducing the allergenic proteins and increasing the proportions of small peptides in SBM with no side products and high reaction rate with mild reaction conditions.^12^ In addition, it has been reported that enzyme-treated SBM can replace antibiotics for reducing diarrhea and improving performance in nursery pigs based on the beneficial effects on antioxidant capacity, immunity, and intestinal barrier function.^13^ Although SBM treatment with enzyme was an effective process, several factors, including long hydrolysis time, alteration of pH during the process, and high reaction temperature, can cause denaturation of the enzyme and affect various properties in the products such as hydrolysate, length, molecular weight, and amino acid composition.^14^ Consequently, the stability of the enzyme should be improved to overcome this limitation.

Enzyme immobilization is a method in which enzymes attach to an inert insoluble material to convert the biological catalysts into reaction catalysts. Immobilization of an enzyme can prevent it from structural denaturation caused by the external environment. Subsequently, enzyme activity can be maintained from various reaction conditions. Enzyme immobilization could be achieved using covalent and noncovalent processes. Covalent bindings using cross-linking agents have been applied for enzyme immobilization onto cellulose fibers from sugar cane bagasses,^15,16^ chitosan materials,^17−21^ clay/chitosan composites,^22,23^ and cellulose ultrafine fibers.^24^ Despite this technique offering high stability of adsorbed enzymes to the environmental conditions such as pH, temperature, ionic strength, and biomolecule concentration, its main disadvantages are the poor knowledge of enzyme structure, the additional purification procedures to eliminate residual toxic reagents, and the lengthy, labor-intensive process. Besides, noncovalent processes including physical adsorption,^25^ encapsulation,^26,27^ and ionotropic gelation^28,29^ have been introduced as alternatives for enzyme stabilizations. Among them, ionotropic gelation (polyelectrolyte complexation) is a simple and mild method with no use of harmful chemicals and elevated temperature for enzyme immobilization based on electrostatic interactions between ions with different charges. On the basis of this technique, it showed that enzyme immobilization onto chitosan nanoparticles by using sodium tripolyphosphate (TPP) as a cross-linking agent could be obtained with immobilization efficiency higher than 84% with loading capacity of 14–16%.^28^ Furthermore, immobilization of enzyme onto katira gum nanoparticles by employing CaCl_2_ or MgCl_2_ as cross-linkers showed high enzyme entrapment of 70% (w/w of katira gum) with a loading capacity of 16%.^29^ It could be concluded that enzyme immobilization based on the principle of ionotropic gelation with the employment of a biocompatible and nontoxic cross-linking agent shows high potential as a promising method to increase enzyme stability for animal feed applications.

**Figure 1:**
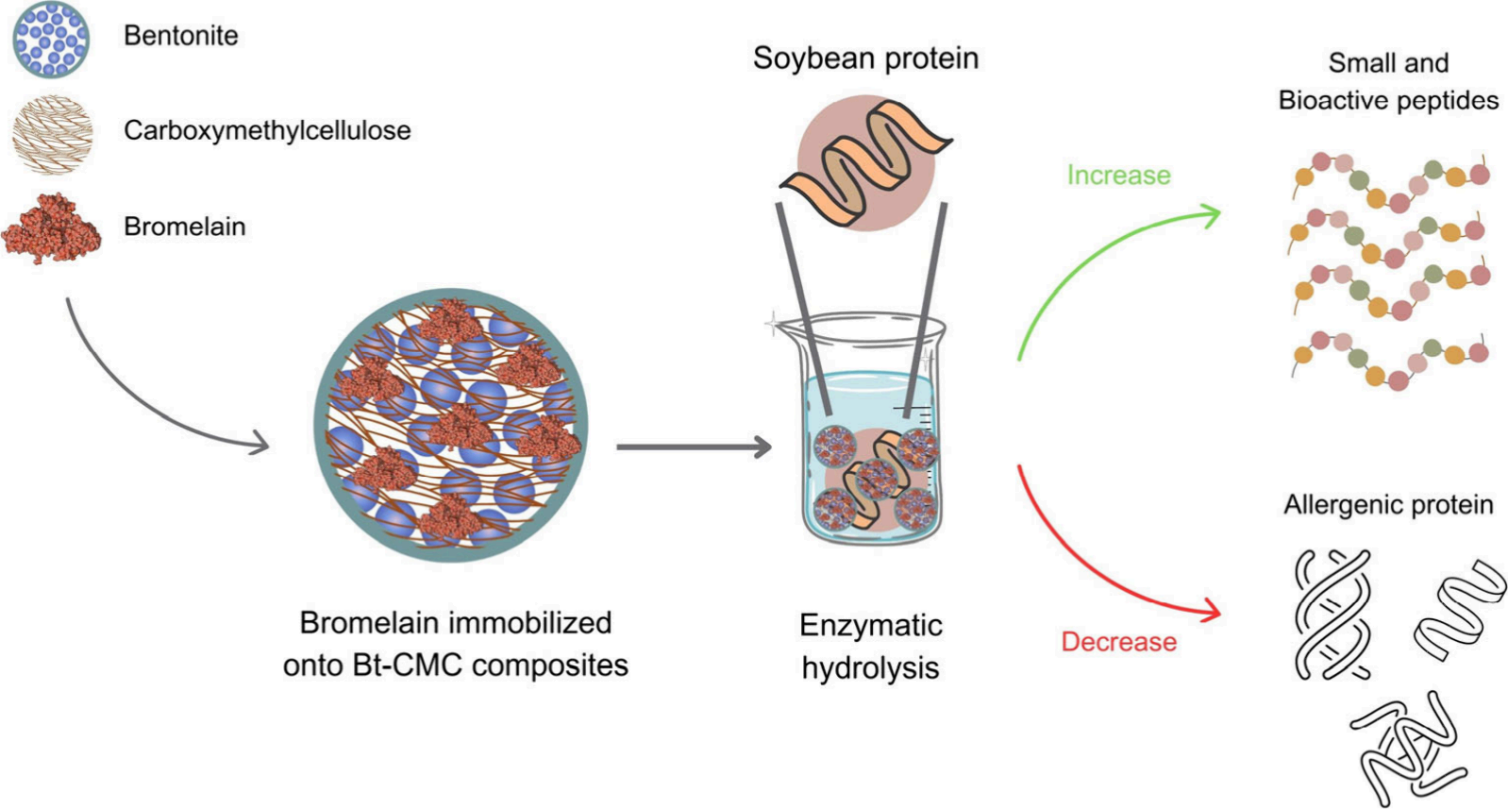
Schematic views of bromelain immobilization onto Bt-CMC composites and application of Br-Bt-CMC composites in the improvement of nutritional values and reduction of allergenic proteins in soybean meal for animal feed application.

In this study, we introduce a bentonite (Bt)-carboxymethylcellulose (CMC) composite as a new material for bromelain (Br) immobilization using an ionotropic gelation technique to produce the Br-Bt-CMC composite. The composite is designed to improve nutritional values through SBM treatment for animal feed applications. This invention was filed for a Thailand petty patent (no. 2303001289) in 2023.^30^ Bromelain is a set of proteolytic enzymes found in the Bromeliaceae family, mainly in pineapple (*Ananas comosus* L.). Bromelain has been widely used in various industries because it is a nontoxic and environmentally friendly cysteine protease for protein hydrolysis with stability over a wide range of pH levels (4.0–8.0) and temperature range of 40–60 °C.^31^ It has been reported that treatment with bromelain could significantly degrade allergenic proteins in SBM used for broilers production^32^ and could effectively produce halal protein hydrolysates from meat and soybean proteins to be as a nitrogen source for the growth of lactic acid bacteria.^33^ CMC contains cellulose groups binding with carboxymethyl groups and hydroxyl groups of the glucopyranose monomers.^34^ It is easily soluble in water and becomes negatively charged. Bentonite (Bt) clay is an abundant and low-cost adsorbent. Bentonite is composed of montmorillonite-based clay, anionic groups which are unbalanced-negative charges of the surface. It contains the “gibbsite layer” between silica layers in the structural unit. This results in the high cation-exchange capacity of bentonite. The presence of negative charges in both CMC and bentonite structures provides a benefit for positively charged amine groups in bromelain to interact with and facilitate composite formation via electrostatic interactions in the presence of calcium cation obtained from CaCl_2_, which is a cost-effective, nontoxic, and environmentally friendly cross-linker.^35^ Calcium is also an important mineral for tissue development, nerve transmission, muscle contraction, blood clotting, osmoregulation, and as a cofactor for the enzymatic procession in animals.^36^ Furthermore, it has been reported that incubation of bromelain with CaCl_2_ could effectively promote the enzyme activity by stabilizing the secondary structure of enzyme. With increasing calcium ion concentrations, enzyme activity was consistently improved.^37^ Consequently, in this study, the Bt and CMC composites are prepared at different CMC to calcium ion (Ca^2+^) mass ratios. Immobilization parameters, enzymatic activity, and structural characterizations are studied. Thermostability, pH sensitivity, and storage stability of the immobilized bromelain are evaluated and compared with that measured from free bromelain. The performance of the immobilized bromelain in SBM treatment is also investigated. Simple schematic views of bromelain immobilization onto Bt-CMC composites as well as the use of Br-Bt-CMC composites in SBM treatment to enhance nutritional values of SBM are summarized in Figure 1.

## 2 Materials and Methods

### 2.1 Materials

Nanoclay [hydrophilic bentonite (Bt)] was purchased from SIGMA-Aldrich (St. Louis, MO, USA). Carboxymethyl cellulose (CMC) was purchased from Union Chemical 1986 Co., Ltd. Bromelain (Br) 2000 GDU (2.91 ± 0.14 U/mg of protein, 46.44% protein content) produced from pineapple stem was supported by Hong Mao Biochemicals Co., Ltd. Calcium chloride anhydrous (Pure-Granular) was purchased from CARLO ERBA. Ninhydrin [1,2,3-indantrione monohydrate, ACS; ≥98.0%(UV)] was purchased from Fluka. Glycine [purity: >99.0%(T)] was purchased from Tokyo Chemical Industry Co., Ltd. Ethanol absolute ≥99.9% was purchased from Merck. Soybean meal was locally purchased at the Bangkok market in Thailand. All solutions were prepared with deionized (DI) water (18.2 M cm of MΩ).

### 2.2 Immobilization of Bromelain onto Clay-Carboxymethyl Cellulose Composites

Immobilization of bromelain onto clay-carboxymethyl cellulose composites was carried out using an ionic gelation technique. First, the suspension of Bt and CMC in DI water was prepared with a Bt:CMC weight ratio of 10:1. After that, the suspension was mixed with a 10% bromelain (Br) solution dissolved in DI water. Then, the mixture was stirred for 30 min and dropped into a 10% calcium chloride solution followed by stirring for 30 min to obtain a Br-Bt-CMC composite with a weight ratio of CMC:Br as 1:1. The composites containing Bt and CMC were prepared at different CMC to calcium ion (Ca^2+^) proportions of 1:10, 1:20, 1:30, 1:40, and 1:50 (w/w). These Br-Bt-CMC composites are called 10, 20, 30, 40, and 50, respectively. The Ca^2+^ solution after composite formation was sampled for further analysis of protein content. The composite was separated and washed several times with DI water. It was dried in a vacuum oven overnight, ground, and kept at 4 °C until use.

### 2.3 Protein Concentration and Enzymatic Activity

Protein concentration of bromelain and other solutions throughout the experiment was determined according to the Bradford method adapted from previous reports using BSA as a standard.^38^ Bromelain enzymatic activity was investigated using assays modified from literature^38^ using casein from bovine milk as substrate, incubated at 37 °C for 10 min. The absorbances of supernatants were measured at 660 nm in a microplate reader (BioTek, Power wave XS2). Enzymatic activity was then calculated in activity units (U/mg solid Br), the amount of enzyme that liberated 1 μmol of tyrosine per minute under the assay conditions.

### 2.4 Determination of Immobilization Parameters and Enzymatic Activity

After immobilization, the percentage of bromelain coupling reported as loading capacity (LC%) and immobilization yield (IY%), were calculated. First, the protein concentrations in the initial bromelain solution and the filtrate were determined. Then, LC% was calculated by using the ratio between the mass of bromelain loaded into composites (*M*_Br_) and the mass of Bt and CMC used for their production (*M*_Bt__+CMC_)), applying eq 1.^38^ The IY% was determined as the difference in bromelain concentration between bromelain in the initial solution (*Br*_initial_) and bromelain in the filtrate (*Br*_filtrate_) as described by eq 2. The enzymatic activity (EA) is measured in units, which indicate the reaction rate catalyzed by that enzyme expressed as micromoles of product formed per minute. In this case, the product is tyrosine.^38^ Therefore, the unit of EA is the unit per milligrams of solid bromelain, as shown in eq 3.
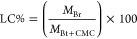
1
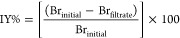
2
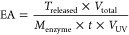
3where *T*_released_ is μmol tyrosine equivalent released, *V*_total_ is total volume of assay (mL), *M*_enzyme_ is mass of enzyme (mg), *t* is time of assay (minute), and *V*_UV_ is volume used in colorimetric determination (mL).

### 2.5 Characterization of Br-Bt-CMC Composites

Chemical functional groups of the produced Br-Bt-CMC composites were investigated using a Fourier Transform Infrared (FT-IR) spectrometer (Thermo Scientific Nicolet iS50), equipped with an attenuated total reflectance (ATR). The scanning range was set from 4000 to 400 cm^–1^ with a resolution of 4 cm^–1^ and 64 scans per sample. X-ray diffraction (XRD) patterns were recorded using Bruker D8 Advance diffractometer equipped with Cu Kα radiation (λ = 0.15418 nm) at 40 kV and 40 mA. The diffraction angle, 2θ, was scanned in a range of 3°–40° with a counting time of 2 s at steps of 0.02°. Basal spacing (*d*_001_) of bentonite clay was calculated according to Bragg’s law following eq 4:^39^
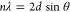
4where *n* is a diffraction order, λ is an incident radiation wavelength (λ = 0.15418 nm), *d* is an interplanar distance or basal spacing, and θ is a diffraction angle.

### 2.6 Enzyme Activities of Free and Immobilized Bromelain in Different pH

The optimum pH of both free and immobilized bromelain was determined by incubating the sample in a 0.65% casein solution prepared in different pH buffers at a temperature of 37 °C. 50 mM phosphate buffers with pH ranging from 3.0 to 8.0 were prepared using 1 M K_2_HPO_4_ and 1 M KH_2_PO_4_. 50 mM Tris HCl buffers with pH ranging from 9.0 to 10.0 were prepared using 1 M Tris Base and 1 M HCl. Bromelain assay was conducted using the method mentioned earlier.

### 2.7 Thermostability of Free and Immobilized Bromelain

The thermostability of free and immobilized bromelain was studied by heating in 50 mM phosphate buffer pH 7.0 at 25, 80, and 100 °C without substrate. Samples were withdrawn after 10 min of the treatment, promptly cooled, and incubated with 0.65% casein solution for 10 min at 37 °C with the subsequent activity determination.

### 2.8 Storage Stability

Storage stability of immobilized bromelain was studied for a period of 10 days. Free and immobilized bromelain were stored in airtight containers and kept in a fridge at 4 °C for comparison. Storage stability test at 25 °C of immobilized bromelain was also conducted as a preliminary study. The samples were packed in vacuum-sealed bags to minimize air and moisture content in the storage environment. On each day, the enzyme activity was determined and used to calculate residual activity by taking activity on the first day as 100%.

### 2.9 Soybean Meal Treatment with Br-Bt-CMC Composites

To improve the nutritional value of soybean meal (SBM), SBM was treated with Br-Bt-CMC composites. The process was carried out by mixing SBM powder (<250 μm of particle size) and immobilized bromelain with a mass ratio of 1.0:0.7, then adding 6 mL of deionized water. The mixtures were incubated at 50, 60, 70, and 80 °C for 30, 60, 90, and 120 min in a shaking water bath with a speed of 120 rpm. After the incubation period, the mixture was boiled at 95 °C for 30 min, followed by adding 4 mL of deionized water. The mixture was then centrifuged at 8000*g* for 10 min at 25 °C. The supernatant was collected for free alpha amino nitrogen (FAN) analysis using the ninhydrin method.^40^ In brief, 1 mL of the supernatant was mixed with 3 mL of DI water, followed by the addition of 1 mL of 2% ninhydrin solution dissolved in ethanol. The mixture was shaken and then heated at 95 °C for 15 min. After cooling for 20 min, 1 mL of 50% ethanol was added to the mixture. FAN was analyzed by comparing with the glycine standard curve using a spectrophotometer at 570 nm and calculated using eq 5:
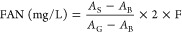
5where *A*_S_ is an average absorbance of sample, *A*_B_ is an average absorbance of a blank value, *A*_G_ is the average absorbance of the glycine standard solution, 2 is the concentration of the glycine standard solution in mg/L, and *F* is a sample dilution factor. The difference in FAN content between SBM and treated SBM was calculated and presented as relative activity of immobilized bromelain. Relative activity of immobilized bromelain (%) = (FAN in treated SBM – FAN in SBM) × 100.

### 2.10 Sample Preparation and Sodium Dodecyl Sulfate-Polyacrylamide Gel Electrophoresis (SDS–PAGE)

The samples for the electrophoresis study were untreated SBM and SBM after treatment with Br-Bt-CMC composite at 60 °C for 30, 60, 90, and 120 min. Sample preparation started by mixing 0.1 g of each sample with 6 mL of deionized water and boiling at 95 °C for 30 min, followed by adding 4 mL of deionized water. The mixture was then centrifuged at 8000*g* for 10 min at 25 °C. The supernatant was collected for interference removal before further proteomics analysis. The process included protein precipitation and desalination. Protein precipitation was performed by drying 200 μL of the sample using a centrifugal concentrator. The dried pellet was resolubilized by adding 100 μL of DI water and mixing with 400 μL of prechilled acetone. The mixture was vortexed and then incubated at −80 °C for 1 h. Precipitated protein was separated by centrifugation at 18,000*g* for 10 min at 4 °C. The protein pellet was resolubilized by urea lysis buffer for protein desalination. Protein desalination was carried out following the manufactured procedure (Thermo Scientific, USA). Briefly, 200 μL of the sample was transferred into a desalting spin column. The flow-through sample was collected, centrifuged at 1000*g* for 2 min, and concentrated using a centrifugal concentrator (TOMY, Japan). The gel electrophoresis experiment was carried out by loading 10 μg of sample onto 12% acrylamide SDS–PAGE using a power supply set at 120 V with a running time of 75 min. After the running period, the gel was washed with DI water and stained with EZBlue Gel Staining Reagent (Sigma-Aldrich, USA) at 4 °C overnight. The gel was destained and washed with DI water until the protein bands were clear. PageRuler Prestained Protein Ladder was used as molecular mass standards. The respective molecular weights and band intensities were recorded for the different samples.

## 3 Results and Discussion

### 3.1 Bromelain Immobilization and Enzyme Activity

**Figure 2:**
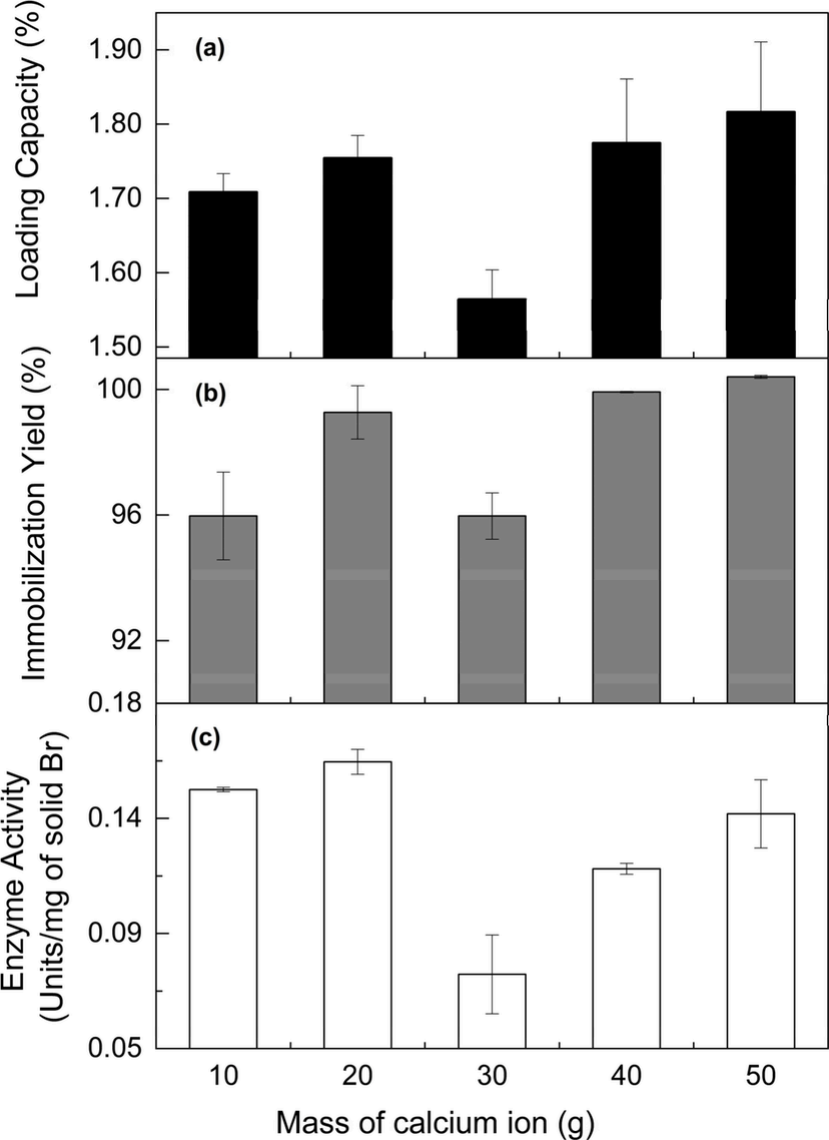
Loading capacity (LC%) (a), immobilization yield (IY%) (b), and enzyme activity (c) of immobilized bromelain prepared in different CMC:Ca^2+^ mass ratios (1:10, 1:20, 1:30, 1:40, and 1:50). The samples were named 10, 20, 30, 40, and 50, respectively.

The effect of calcium ion contents during composite formation on bromelain (Br) immobilization was studied by considering LC%, IY%, and EA. Figure 2a shows the loading capacity (LC%) of bromelain onto Bt-CMC composites using different mass ratios of CMC: Ca^2+^ (1:10, 1:20, 1:30, 1:40, and 1:50). All samples revealed LC% in the range of 1.5–1.8%. Among them, a ratio of 1:50 provided the highest LC% (1.82 ± 0.09%), while a ratio of 1:30 showed the lowest LC% (1.56 ± 0.04%). It was noted that the lowest LC% when using a ratio of 1:30 was likely caused by effect of net charge equilibrium in the suspension, which exhibited low attraction for bromelain to interact with and form the composite. The LC% value obtained from this study was relatively low compared to that reported using the ionotropic gelation technique (14–16)^28,29^ and also other technologies such as covalent immobilizations (2–68%),^16,20,24^ encapsulation processes using double emulsion solvent evaporation methods (4–5%),^27,41^ and absorption process (7–8%).^42^ For industrial applications, high enzyme loading capacity is important as it can increase the process efficiency and reduce material quantity as well as material cost. However, the composite with low enzyme content is beneficial for the application in soybean meal (SBM) treatment in this study. This is because the treatment requires a large quantity of composite materials to allow mixing well and dispersion of the enzyme in SBM matrix and to enable an effective hydrolysis process. Immobilization yield was further determined, as shown in Figure 2b. All samples exhibited IY% higher than 95%. The enzyme activity (EA) of all samples was investigated, as presented in Figure 2c. EA of all samples was found in a range of 0.07–0.16 U/mg of solid Br. The highest EA was observed from the ratio of 1:20 (0.16 ± 0.01 U/mg of solid Br), while the lowest EA was found at a ratio of 1:30 (0.07 ± 0.02 U/mg of solid Br). The Br-Bt-CMC composite prepared using a CMC:Ca^2+^ mass ratio of 1:20 was further used for the leaking test in water at 25 °C. Less than 1% of enzyme leaking was found after placing it in water for 30 min (see Figure S1). In conclusion, bromelain could be successfully immobilized onto Bt-CMC composites using an ionotropic gelation technique with a high immobilization yield of more than 95% and less leaking in water.

### 3.2 Characterization of Bromelain Immobilized onto Bt-CMC Composites

**Figure 3:**
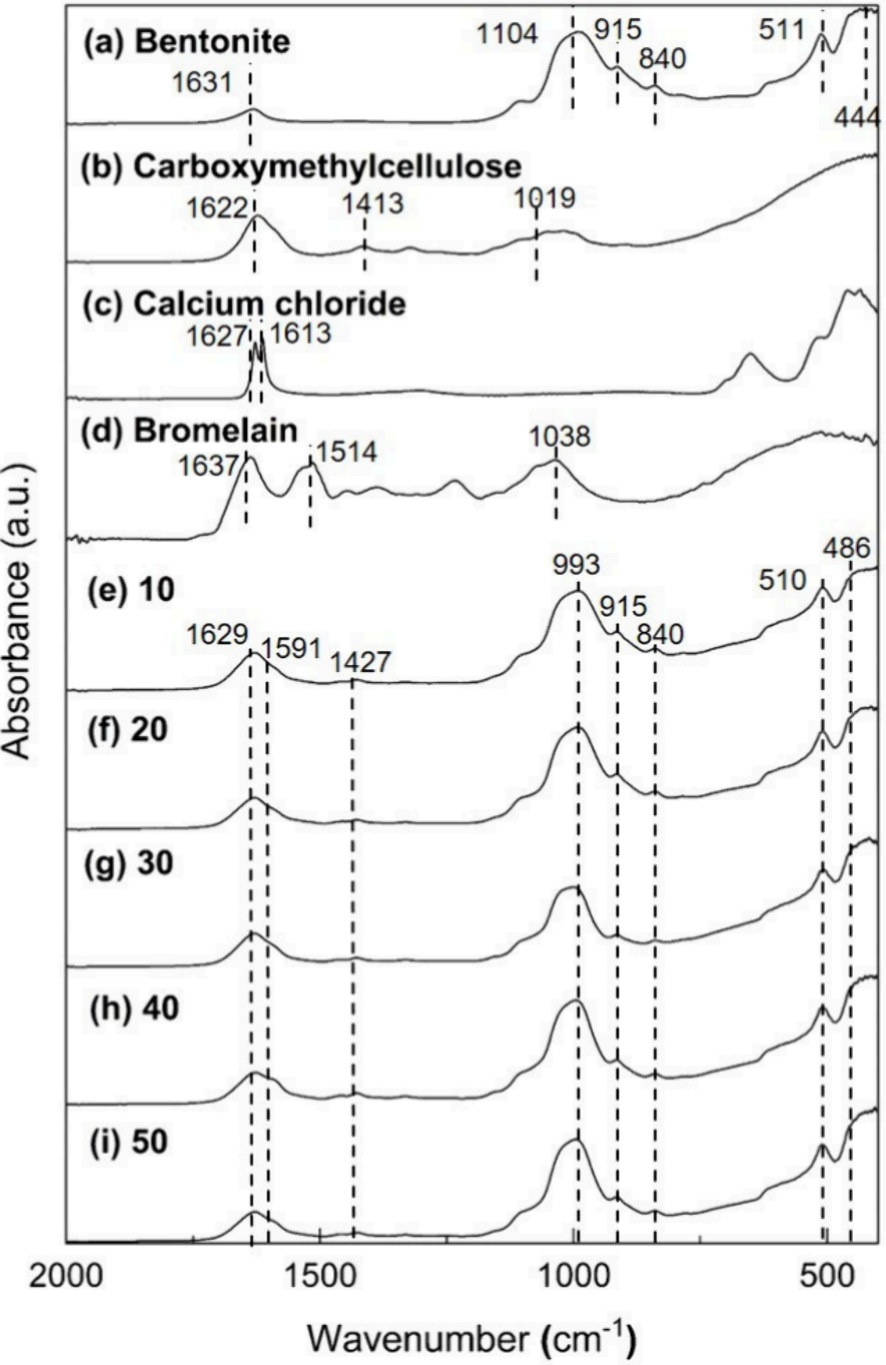
FTIR spectra of bentonite (a), CMC (b), calcium chloride (c), bromelain (d), and Br-Bt-CMC composites with calcium ion ratios of 10 (e), 20 (f), 30 (g), 40 (h), and 50 (i), in a region of 400–2000 cm^–1^.

**Figure 4:**
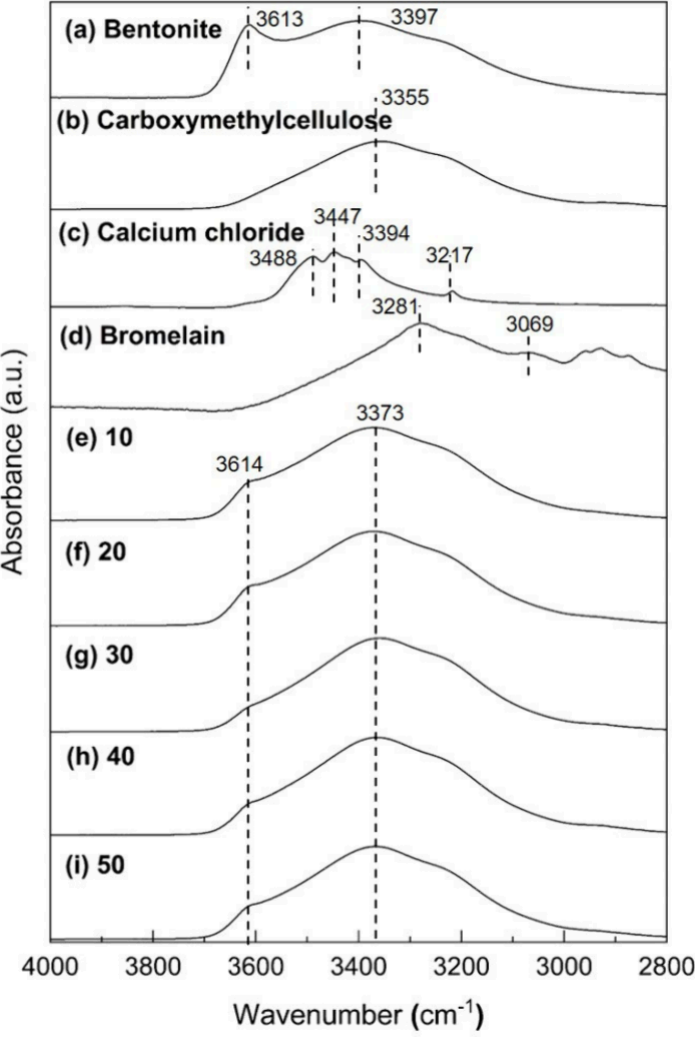
FTIR spectra of bentonite (a), CMC (b), calcium chloride (c), bromelain (d), and Br-Bt-CMC composites with calcium ion ratios of 10 (e), 20 (f), 30 (g), 40 (h), and 50 (i), in a region of 2800–4000 cm^–1^.

The chemical structures of bromelain immobilized onto Bt-CMC composites at different CMC:Ca^2+^ mass ratios (1:10, 1:20, 1:30, 1:40, and 1:50) were analyzed using FTIR spectroscopy, whose spectra in two regions are compared: 400–2000 cm^–1^ (Figure 3) and 2800–4000 cm^–1^ (Figure 4). The raw materials, including bentonite, CMC, calcium chloride, and bromelain, were first analyzed. The spectrum of bentonite (Figure 3a) revealed peaks at 444, 511, and 1104 cm^–1^, assigned to Si–O–Si, Al–O–Si, and Si–O stretching modes of the tetrahedral layer, respectively.^43^ The bands in an 840–915 cm^–1^ region and a peak at 1631 cm^–1^ were attributed to the OH bending of the octahedral layer.^44^ The band at 1104 cm^–1^ was contributed to the Si–O stretching mode of the tetrahedral layer.^43^ CMC exhibited three main peaks at 1019, 1413, and 1622 cm^–1^, associated with C–O bending, CH_2_ scissoring, and C=O stretching mode of the COO– group, respectively.^45^ The spectrum of calcium chloride showed a broad band at 1613–1627 cm^–1^ of the H–O bending mode of water, indicating its hygroscopic nature.^46^ Bromelain displayed absorption bands in a range of 1514–1637 cm^–1^, consisting of overlapped amide I and amide II bands.^47^ After bromelain immobilization, the spectra of all composites (Figure 3e–i) were dominated by the characteristic bands of bentonite due to its relatively high absorptivity and low enzyme content on the Br-Bt-CMC composite. A weak band at 1427 cm^–1^ associated with CH_2_ scissoring and a small shoulder around 1591 cm^–1^ corresponding to the COO– group in the CMC structure were observed. This shoulder band became more prominent with increasing Ca^2+^ content in the composite formation. This is likely caused by an increasing number of water molecules that are bound to calcium ions.

The spectrum of bentonite (Figure 4a) showed board band around 3397 cm^–1^ with a spike peak at 3612 cm^–1^ corresponding to the O–H stretching modes of bound water and the hydroxyls in the clay structures, respectively.^44^ This reflects the presence of water molecules trapped in the bentonite structures before composite formation. CMC exhibited a broad O–H stretching band centered at 3355 cm^–1^, which is characteristic of its abundant hydroxyls.^45^ Calcium chloride showed a band at 3217 cm^–1^ corresponding to the symmetric O–H stretching and at 3394, 3447, and 3488 cm^–1^ attributed to asymmetric O–H stretching modes of bound water.^46^ Bromelain displayed relatively sharper bands covering a lower wavenumber region at 3069–3281 cm^–1^ due to its O–H and N–H stretching modes, which are strongly hydrogen bonded.^48^ After bromelain immobilization, the spectra of all composites (Figure 4e–i) were dominated by the characteristic bands of bentonite and CMC, as these are the major components of the composites. Nevertheless, the results confirm that Br-Bt-CMC composites were successfully formed using ionotropic gelation in the presence of calcium ions.

**Figure 5:**
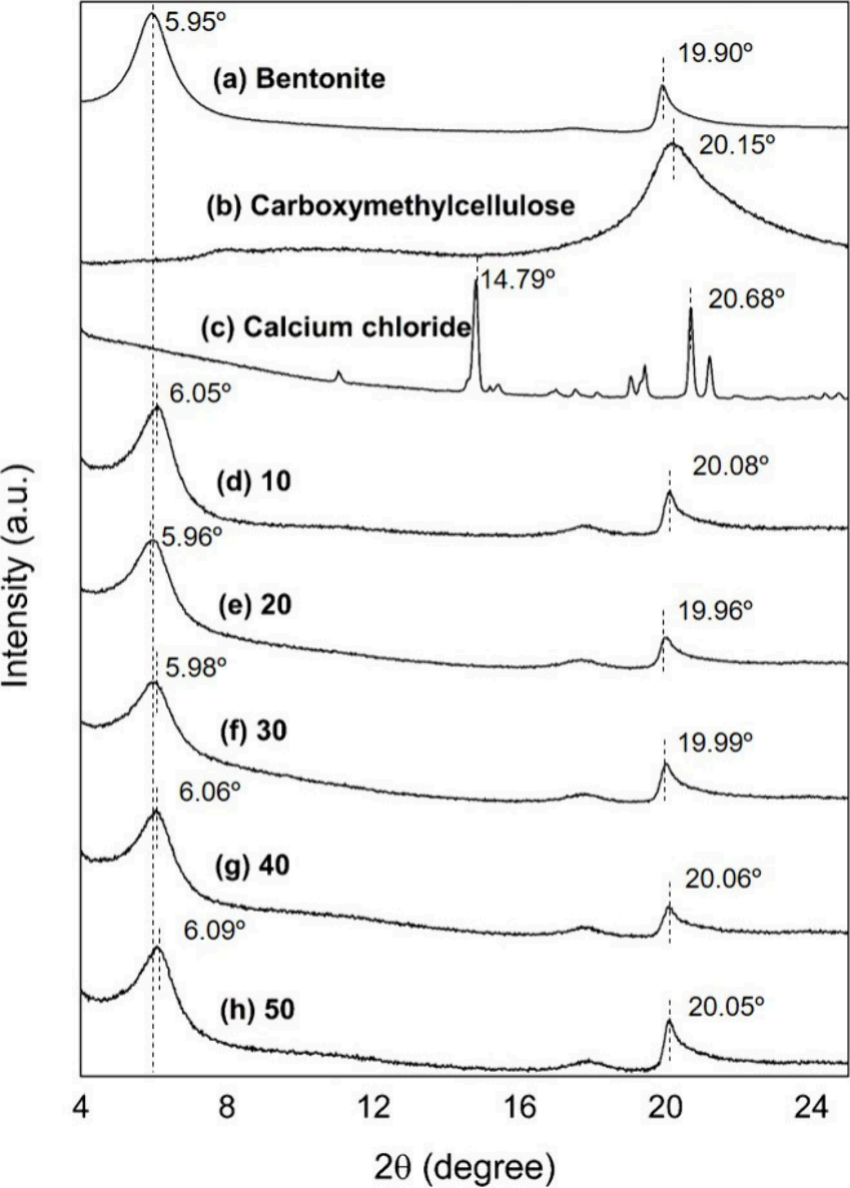
XRD patterns of bentonite (a), CMC (b), calcium chloride (c), and Br-Bt-CMC composites with calcium ion mass ratios of 10 (d), 20 (e), 30 (f), 40 (g), and 50 (h) in a 2θ angle with the region of 4°–25°.

**Figure 6:**
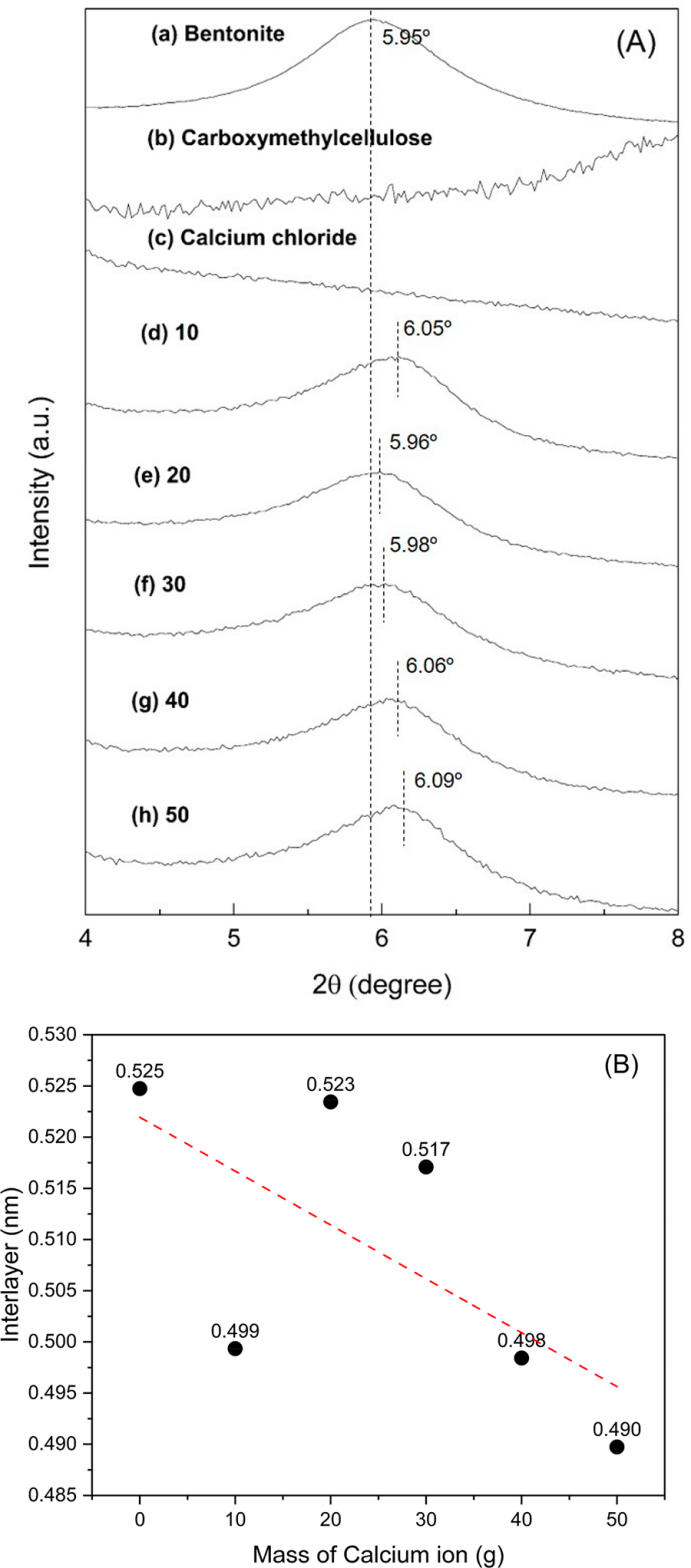
XRD patterns (A) of bentonite (a), CMC (b), calcium chloride (c), and Br-Bt-CMC composites prepared at different CMC:Ca^2+^ mass ratios (1:10, 1:20, 1:30, 1:40, and 1:50) in the 2θ region of 4–8°. The samples were named (d) 10, (e) 20, (f) 30, (g) 40, and (h) 50, respectively. Interlayer spacing (B) of bentonite and Br-Bt-CMC composites prepared at different CMC:Ca^2+^ mass ratios. The dashed line in the graph indicates the trend of all data.

XRD patterns of bromelain immobilized onto Bt-CMC composites prepared at different CMC: Ca^2+^ mass ratios (1:10, 1:20, 1:30, 1:40, and 1:50) were analyzed in 2 theta ranges of 3°–25° (Figure 5) and 4°–8° (Figure 6A). Figure 5a demonstrates that the sample displays well-defined crystallization with a predominant montmorillonite structure, revealing distinctive characteristic features at peak 2θ = 5.95° and 19.90°, which are attributed to *d*_020_ and *d*_001_ basal spacings of the montmorillonite, respectively. The observed basal spacing of *d*_001_ = 19.90° indicates the presence of sodium, thereby allowing the characterization of the raw material primarily as sodium bentonite (Na-bentonite).^49^ XRD pattern of CMC in Figure 5b displayed a main peak at 2θ = 20.15°, corresponding to amorphous regions and small crystallites within the cellulose granules.^50^ In Figure 5c, peaks observed at 14.79° and 20.68° are associated with calcium chloride.^51^ After bromelain immobilization, the XRD patterns of all Br-Bt-CMC composites (Figure 5d–h) were dominated by peaks of montmorillonite. The changes in the clay’s interlayer spacings with an increase in the Ca^2+^ mass ratio was monitored by considering the basal spacing value (*d*_001_). This was calculated by Bragg’s eq (eq 4) using the peaks at 5°–6°, corresponding to the 001 reflection (Figure 6A). Taking into account the thickness of the silicate layer (approximately 0.96 nm), an increase in the interlayer distance in each composite is calculated and plotted as a function of Ca^2+^ mass increase, as shown in Figure 6B. The interlayers of all samples were in the range of 0.490–0.525 nm. Pristine Na-bentonite clay has an interlayer distance of 0.525 nm, which was caused by the presence of hydration shells of Na^+^ in the second-layer (2W) hydrate states.^52^ After composite formation, the interlayer spacing significantly decreased as Ca^2+^ content in the composite increased and reached the value of 0.490 nm when using a mass ratio of CMC:Ca^2+^ of 1:50. This was caused by the replacement of hydrated Na^2+^ ions by hydrated Ca^2+^ ions, which were distributed in the interlayer in the 2W hydration state.^52^ In conclusion, the XRD results provided confirmation for Br-Bt-CMC composite formation and indicated that calcium ion content strongly affected the interlayer spacing of bentonite clay.

### 3.3 Thermostability of Free and Immobilized Bromelain

**Figure 7:**
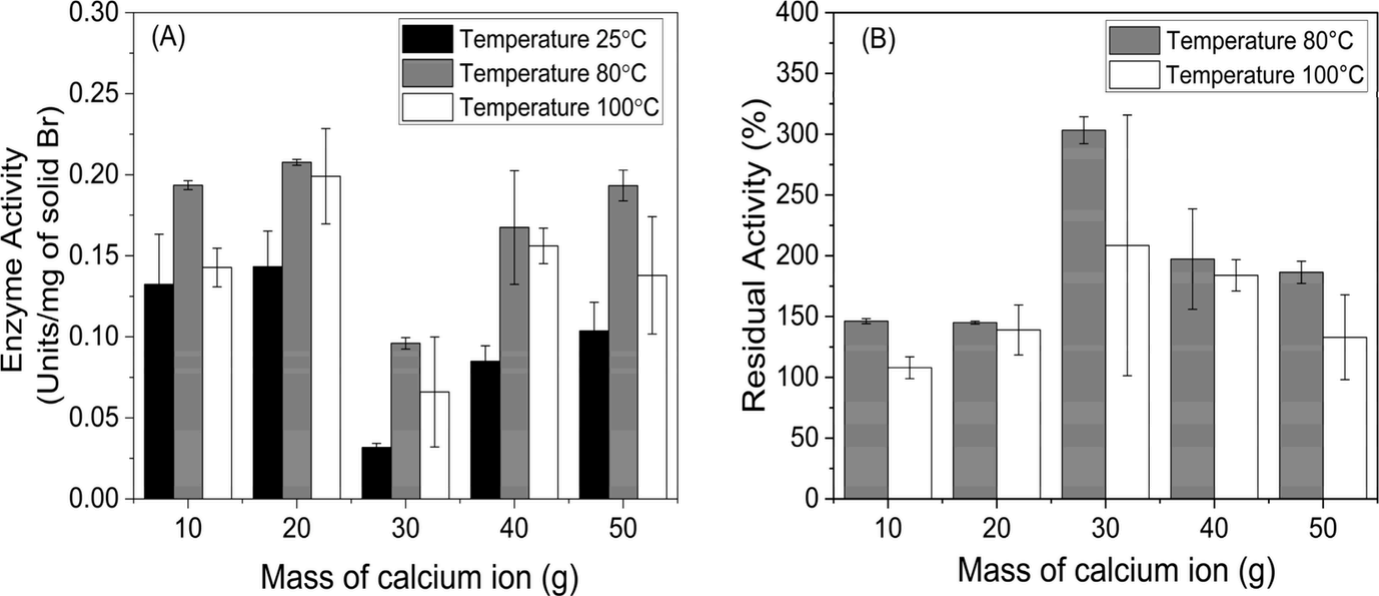
Enzyme activity (A) and residual activity (B) of immobilized bromelain prepared in different CMC:Ca^2+^ mass ratios (1:10, 1:20, 1:30, 1:40, and 1:50). The samples were named 10, 20, 30, 40, and 50, respectively. The samples were treated at 25, 80, and 100 °C for 10 min.

The thermal stability of the composites was evaluated by heating them in 50 mM phosphate buffer pH 7.0 at 80 and 100 °C in the absence of substrate for 10 min. The results were compared with those obtained at 25 °C. Figure 7A showed that all high-temperature tests had less of an inactivation effect on immobilized enzymes in all composites. In most cases, the high temperature could obviously improve the enzyme activity, as found in the residual activity of all samples (Figure 7B), which was higher than 100%. This improvement was possibly the result of a stimulatory effect produced by the presence of calcium ion (Ca^2+^) in the solid matrix near the active sites of the bromelain. While in the same condition at 100 °C, free bromelain lost most of its activity after 10 min and presented residual activity of 0.96 ± 0.19% from its original activity (data not shown). After treatment, the Br-Bt-CMC composite with a CMC:Ca^2+^ ratio of 1:20 exhibited superior thermostability in both temperatures with high enzyme activities of 0.21 ± 0.01 and 0.20 ± 0.03 U/mg of solid Br and high residual activity ∼140%.

### 3.4 Effect of pH on Bromelain Activity

**Figure 8:**
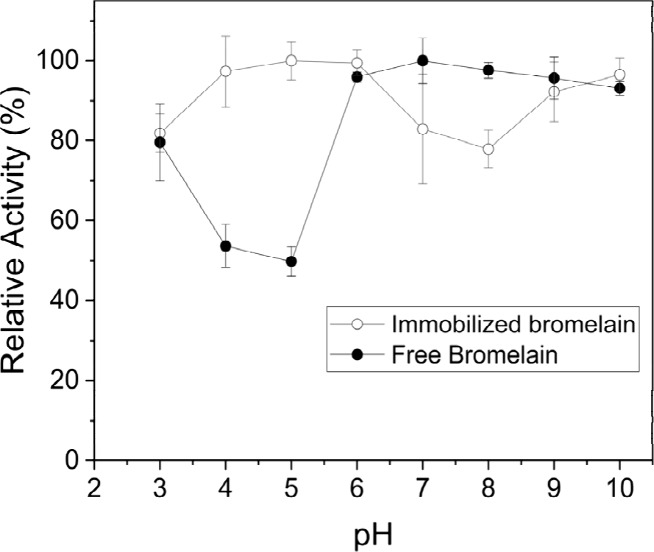
Effect of pH on enzyme activity of free and immobilized bromelain.

The results on the effects of pH in a range of 3–10 on activity of both free and immobilized bromelain are shown in Figure 8. Bromelain immobilized on Br-Bt-CMC composites with a mass ratio of 1:20 was used in this experiment. The relatively high activity (>80%) was found in the pH range of 6–10 for free bromelain and 3–6 for the immobilized bromelain. For the present result, it should be noticed that the immobilized enzyme seems to show higher activity in an acidic rather than in an alkaline condition. The optimum activity of free bromelain was found at pH 7.0, while the immobilized bromelain showed optimum activity at pH 5.0. The shift of optimum pH toward the acidic side of the immobilized bromelain compared to free bromelain was caused by the influence of the charge property of the solid support, which is near the active sites of the bromelain.^53^ In this study, the presence of calcium ion (Ca^2+^), possessing a positive charge, in the matrix obviously affects the optimum pH of the immobilized bromelain to shift to lower values, which is in agreement with a previous report.^25^ It was noticed that the activities were significantly decreased in an acidic pH range with the lowest activity at pH 5 for free bromelain and in an alkaline pH range with the lowest activity at pH 8.0 for the immobilized bromelain. Low enzyme activities at these pH values was caused by a decrease in electrostatic interaction between the enzyme and substrate resulting in unfavorable changes in enzyme or substrate conformation and decrease in enzyme flexibility under drastic changes in pH.^54^ The shift of pH at lowest activity from the alkaline side of the immobilized bromelain compared to free bromelain was caused by the influence of the charge property of the solid support containing Ca^2+^.

### 3.5 Storage Stability

**Figure 9:**
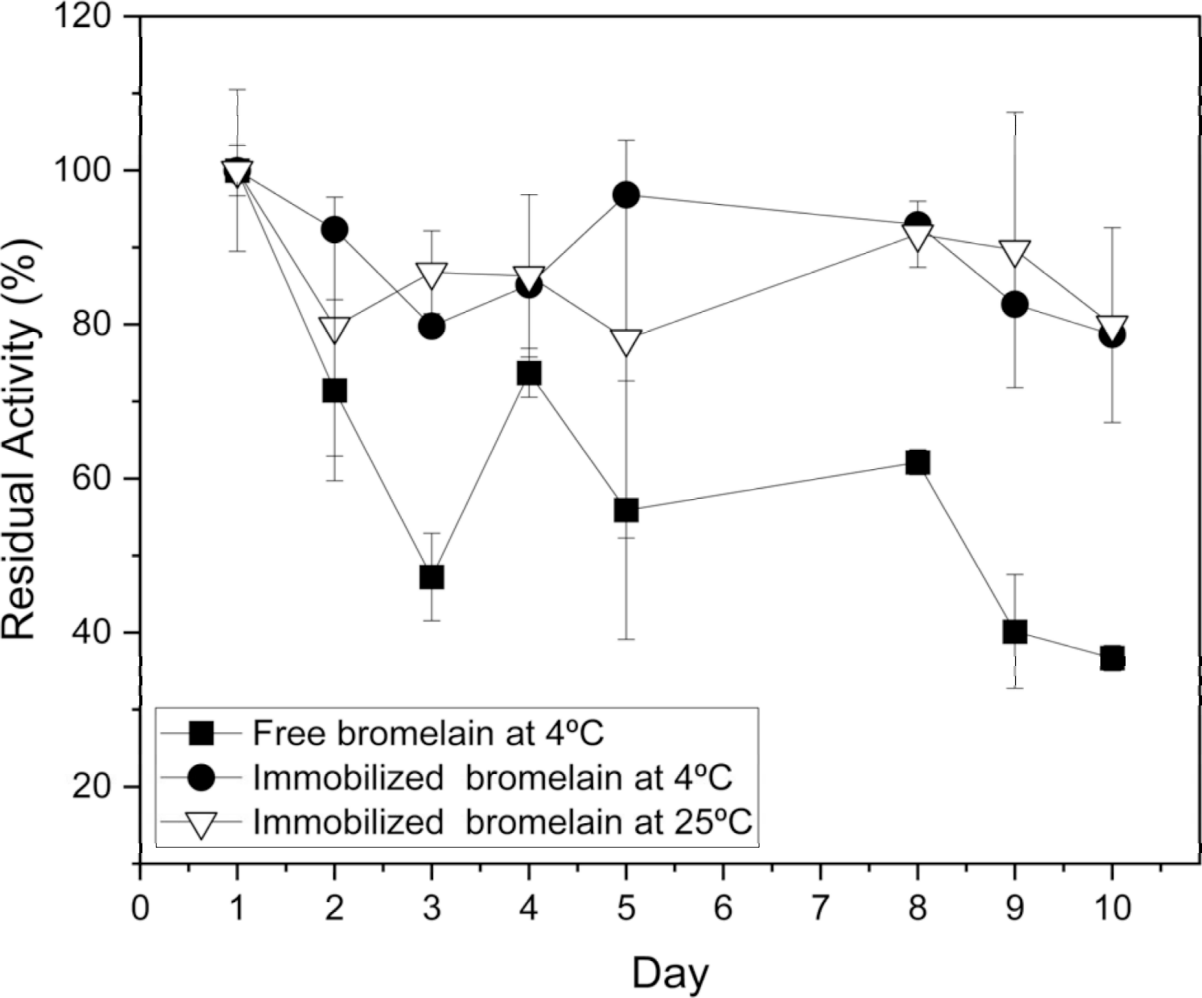
Storage stability of free and immobilized bromelain at 4 °C and immobilized bromelain stored in a vacuum seal bag at 25 °C.

The storage stability of immobilized bromelain was examined by following the residual activity of the enzyme for a period of 10 days, as shown in Figure 9. Bromelain immobilized on Bt-CMC composites with a CMC:Ca^2+^mass ratio of 1:20 was used in this experiment. Free and immobilized bromelain were stored in airtight containers and kept in a refrigerator at 4 °C for comparison. The free bromelain lost its activity continuously over the storage period and reached 36.70 ± 1.63% of residual activity. At the same time, the immobilized bromelain showed residual activity of 78.70 ± 5.59% after 10 days. This suggests that immobilization of bromelain on Bt-CMC composites could significantly enhance the enzyme’s tolerance stress to the environment, leading to improvement in its storage stability. The immobilized bromelain was further tested by storage at 25 °C in vacuum-sealed bags with low moisture. The immobilized bromelain was remarkably stable and could retain its residual activity of 79.91 ± 12.64% on the tenth day. Thermal stability of the immobilized bromelain over storage duration could be synergistic effects of (i) structure stabilization of the dried enzyme by Bt-CMC in the composite matrix and (ii) low moisture in the storage environment that could facilitate desiccation and minimize hydrolysis reaction, which causes autolysis of the enzyme. This remarkable stability offers more advantages over the conventional storage condition, which requires a freezer at temperatures in the region from −10 to −25 °C, in terms of low-cost storage and shipping conditions, enabling its practical utilization in industries.

### 3.6 Soybean Meal Treatment with Immobilized Bromelain

**Figure 10:**
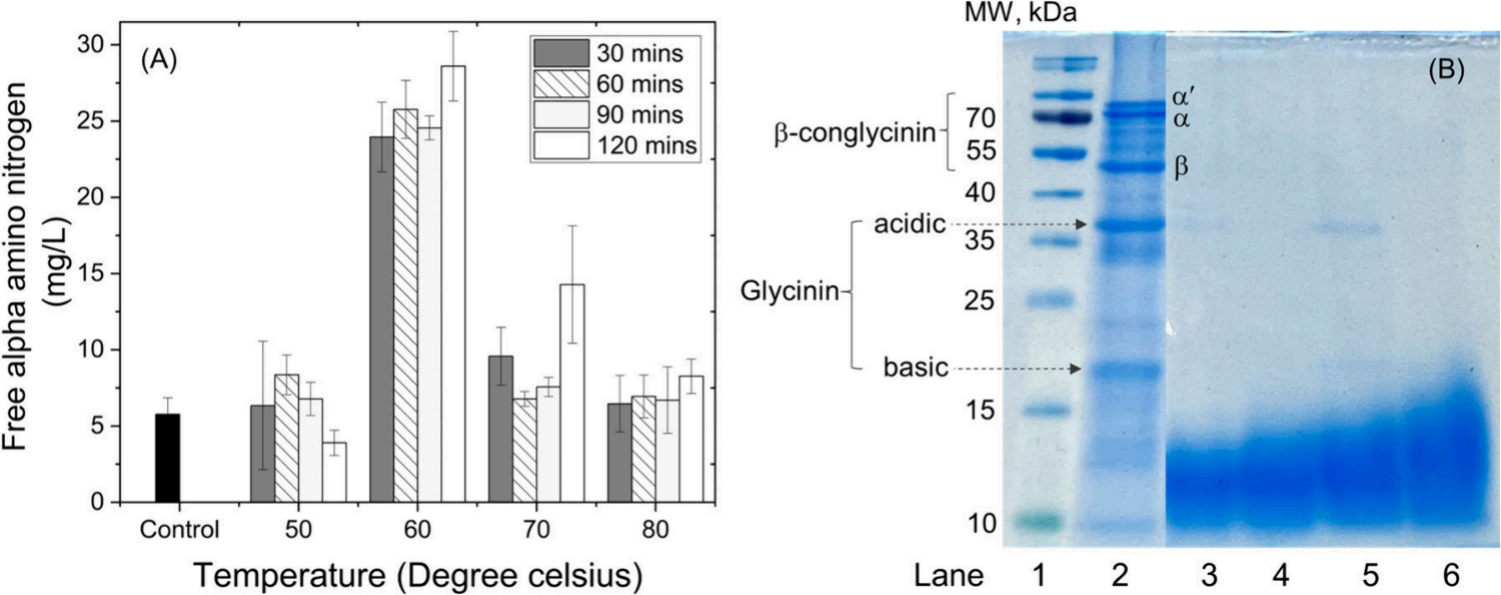
(A) Free alpha-amino nitrogen (FAN) produced from soybean meal treatment with immobilized bromelain at different temperatures (50, 60, 70, and 80 °C) for 30, 60, 90, and 120 min. (B) SDS–PAGE profiles of molecular weights of standard protein (lane 1), SBM protein subunits (lane 2), SBM after treatment with immobilized bromelain at 60 °C for 30 (lane 3), 60 (lane 4), 90 (lane 5), and 120 min (lane 6).

The performance of immobilized bromelain for nutritional value improvement of soybean meal (SBM) was investigated. Bromelain immobilized on Bt-CMC composites with a CMC:Ca^2+^mass ratio of 1:20 was used for this purpose due to its superior thermostability with high enzyme activity. Efficiencies of SBM hydrolysis using the immobilized bromelain at different temperatures (50, 60, 70, and 80 °C) and various times (30, 60, 90, and 120 min) were carried out by following concentrations of free alpha amino nitrogen (FAN), which is a product from hydrolysis of SBM by the enzyme. As shown in Figure 10A, the results reveal that treatment SBM at 60 °C presented the highest FAN concentrations (23.96–28.60 mg/L) for all treatment periods. This nutritional value was increased by ∼4–5 times from that presented in untreated SBM (5.77 ± 1.08 mg/L). Whereas, treatments at 50, 70, and 80 °C did not show significant improvement in FAN concentrations (3.90–14.28 mg/L) compared to that observed in untreated SBM. This suggests that 60 °C is an optimum temperature that provides suitable activation energy for the reorganization of the immobilized bromelain molecules to the conformation necessary for hydrolysis to occur. Protein profiles of the samples treated at 60 °C for 30, 60, 90, and 120 min were further analyzed by SDS–PAGE compared with untreated SBM, as shown in Figure 10B. The main components of SBM protein, including α′, α, and β subunits of β-conglycinin (78, 70, and 47 kDa) and glycinin acidic and basic subunits (37 and 19 kDa), respectively, were identified from lane 2.^55,56^ These glycinin and β-conglycinin are known as two major allergenic proteins in SBM.^3^ After treatment, electrophoresis lanes obtained from all treated SBM showed mostly a large number of proteins <25 kDa with a few shadow bands of the 37 kDa glycinin acidic and 19 kDa glycinin basic subunits. This implies that most proteins with molecular weight higher than 25 kDa, including β-conglycinin, were degraded. It was noticed that a longer hydrolysis time tended to produce a higher proportion of large peptides. This is possibly because our substrate, SBM, contains several proteins of various sizes resulting in more types of proteins for digestion and yielding larger peptides at longer incubation time. In addition, partial denaturation of the immobilized enzyme could potentially occur during the reaction process leading to incomplete protein digestion and increase in proportion of large molecular mass of peptides (see Figure S2). The molecular mass distribution of peptide obtained from these samples were determined using MALDI-TOF mass spectrometry (see Figure S3). This reveals that all treated conditions of SBM exhibited the same mass fingerprints distributed in a range lower than 1800 *m*/*z*. The results indicate that soy proteins in SBM were effectively broken down into small peptides with high selectivity by treatment with the Br-Bt-CMC composite within 30 min, which is shorter than that required for the microbial fermentation process (>24 h). On the basis of these results, it could be concluded that the Br-Bt-CMC composites could effectively speed up the degradation of soybean proteins, improvement of nutritional value, and allergenicity reduction in SBM with high selectivity.

## 4 Conclusions

In conclusion, bromelain enzyme was successfully immobilized onto bentonite-carboxymethylcellulose composite through the ionotropic gelation method. On the basis of this technique, immobilization yields higher than 95% and remarkedly high thermal stability of the enzyme were obtained with less leakage. Among all samples, a composite with the CMC:Ca^2+^ ratio of 1:20 exhibited superior thermostability at 100 °C with high enzyme activity of 0.20 ± 0.03 U/mg of solid enzyme and high residual activity of 139 ± 20% from its original activity. XRD results indicate that the replacement of hydrated Na^2+^ ions by Ca^2+^ ions in the interlayer of bentonite clay occurred during the immobilization process. This resulted in a reduction of interlayer spacing of the clay with an increase in the Ca^2+^ content in the preparation environment. The presence of Ca^2+^ in the Br-Bt-CMC composite structures significantly affected the charge property surrounding the active sites of the immobilized bromelain, causing the shift of optimum pH for enzyme activity to the acidic side. The Br-Bt-CMC composites could enhance the enzyme’s tolerance to stress in the environment, improving storage stability. The use of immobilized bromelain for SBM treatment was also studied. It showed that the composite could effectively hydrolyze SBM to increase the nutritional value up to ∼4 times and lower allergenic proteins from that presented in untreated SBM within 30 min.

## Supporting Information Available

Details regarding protein leakage (%) of free bromelain and immobilized bromelain at 25 °C at 0, 10, and 30 min in water; mass spectra of SBM after treatments with immobilized bromelain at 60 °C for 30, 60, 90, and 120 min; and measurements were carried out in a linear mode, positive ionization, and mass ranges: 750–3000 *m*/*z* and 10,000–200,000 *m*/*z* (PDF)
