# Supplementary material for: Bromelain Immobilized onto Clay-Carboxymethylcellulose Composites for Improving Nutritive Value of Soybean Meal
Source: ACS Appl Bio Mater. 2024 Jul 18;7(8):5211–21. doi: 10.1021/acsabm.4c00392 (PMC11337166; doi:10.1021/acsabm.4c00392)
Supplement: Supplementary file 1 — mt4c00392_si_001.pdf [file mt4c00392_si_001.pdf]

Supporting Information

Bromelain Immobilized onto Clay-Carboxymethylcellulose Composites for Improving Nutritive Value of Soybean Meal

Kanlayanit Pimcharoen[a], Pakorn Opaprakasit[a], Yodying Yingchutrakul[b], Nattapon Simanon[b], Chutikarn Butkinaree[b], Darawan Yuttayong[c], Ramawadee Hompa[d], Lapporn Vayachuta[d], Panida Prompinit[d, *]

[a]School of Integrated Science and Innovation, Sirindhorn International Institute of Technology (SIIT), Thammasat University, Pathum Thani 12121, Thailand
[b]National Center for Genetic Engineering and Biotechnology, National Science and Technology Development Agency (NSTDA), Khlong Luang, Pathum Thani, 12120, Thailand
[c]Aquatic Animal Feed Research and Development Division, Department of Fisheries, Ministry of Agriculture and Cooperatives, Bangkok, 10900, Thailand.
[d]National Nanotechnology Center (NANOTEC), National Science and Technology Development Agency (NSTDA), Khlong Luang, Pathum Thani, 12120, Thailand

Correspondence
Panida Prompinit, National Nanotechnology Center (NANOTEC), National Science and Technology Development Agency (NSTDA), Khlong Luang, Pathum Thani, 12120, Thailand.
Email: panida@nanotec.or.th

1. Enzyme leaking test

The immobilized enzyme protein leaking from the composites into water was determined with the following method: 0.01 g of immobilized bromelain was added into 1 ml DI water and incubated at 25℃. Samples were withdrawn at different intervals (10, 20, and 30 minutes), followed by protein determination using the Bradford method. The enzyme leakage at each time interval was determined as the ratio of leaked protein to the total protein immobilized on the support.

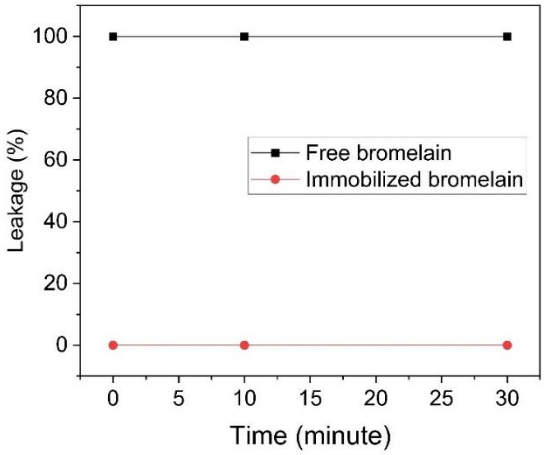

Figure S1 Protein leakage (%) of free bromelain and immobilized bromelain at 25℃ at 0, 10, and 30 minutes in water.

2. Mass spectrometry analysis of soybean meal and treated soybean meal

To analyze the low molecular mass distribution using mass spectrometry, a desalting technique was performed to remove contamination during mass spectrometric acquisition using Pierce™ C18 Tips (Thermo Scientific, USA) following the manufacturer's procedures. Briefly, C18 tip was wetted by 50% acetonitrile (ACN) and equilibrated with 0.1% trifluoroacetic acid (TFA). The sample was loaded into the C18 tip and washed with 5% ACN containing 0.1% TFA. Then it was eluted by 60% ACN containing 0.1% TFA, followed by mixing with α-cyano-4-hydroxycinnamic acid (α-CHCA), which was used as a matrix for laser desorption/ionization at a ratio 1:1 of sample per matrix. Subsequently, 1 µl of the mixture was deposited onto the target plate and dried at room temperature before loading into the MALDI-TOF mass spectrometer. MALDI-TOF signals were acquired using msTornado Control software (JEOL, Japan) with the following parameters: positive polarity, linear mode, mass range between 700 to 3,000 m/z, sampling interval 0.5 ns, 47% laser intensity, laser frequency 250 Hz, high voltage target plate 20 kV. A mass spectrum was exported to msTornado Analysis software (JEOL, Japan).

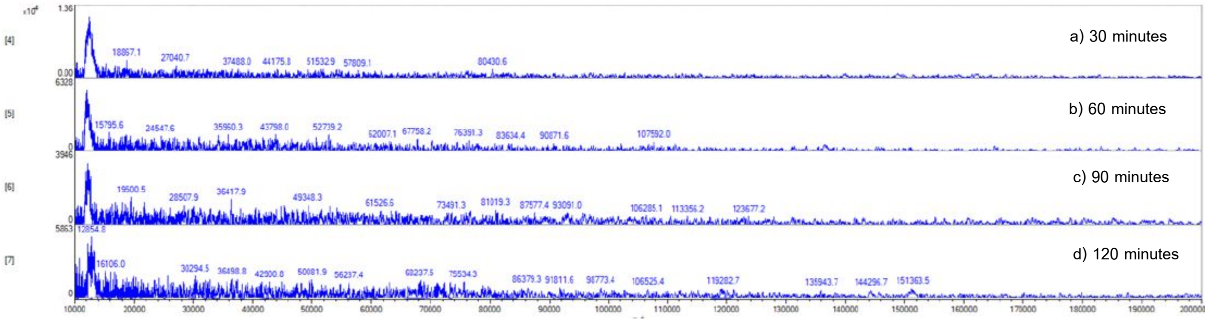

Figure S2. Mass spectra of SBM after treatments with immobilized bromelain at 60°C for 30, 60, 90, and 120 minutes. The measurements were carried out in a linear mode, positive ionization, mass range: 10,000-200,000 *m/z*.

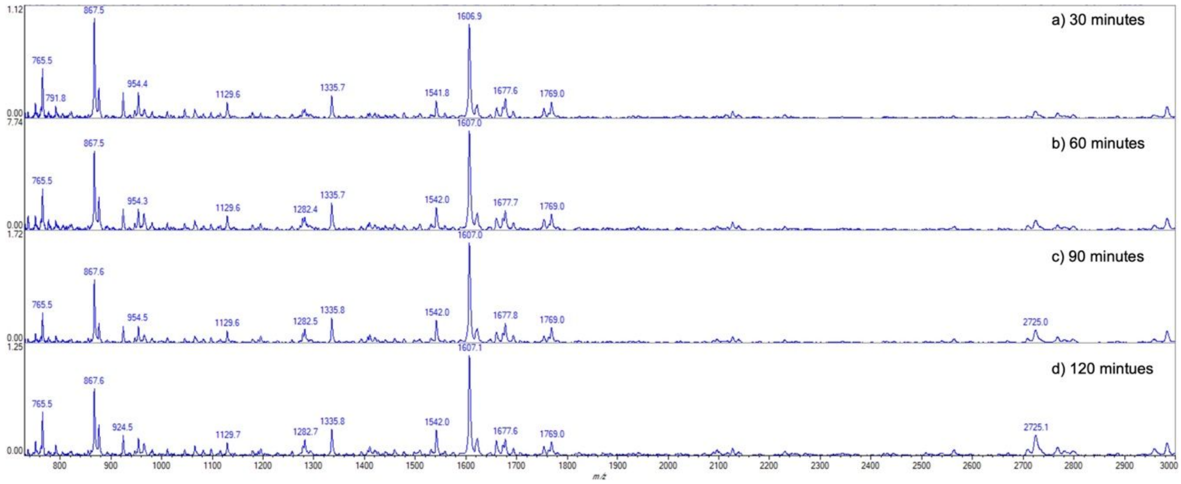

Figure S3. Mass spectra of SBM after treatments with immobilized bromelain at 60°C for 30, 60, 90, and 120 minutes. The measurements were carried out in a linear mode, positive ionization, mass range: 750-3,000 *m/z*.
